# Effects of Histamine and the α-Tocopherol Metabolite α-13′-COOH in an Atopic Dermatitis Full-Thickness Skin Model

**DOI:** 10.3390/molecules28010440

**Published:** 2023-01-03

**Authors:** Rebecca Riedl, Maria Wallert, Stefan Lorkowski, Cornelia Wiegand

**Affiliations:** 1 Department of Dermatology, University Hospital Jena, 07743 Jena, Germany; 2 Institute of Nutritional Science, Friedrich Schiller University Jena, 07743 Jena, Germany; 3 Competence Cluster for Nutrition and Cardiovascular Health (nutriCARD) Halle-Jena-Leipzig, 07743 Jena, Germany

**Keywords:** histamine, atopic dermatitis full-thickness skin model, hyperkeratosis, α-13’-carboxychromanol, anti-inflammatory, anti-allergic

## Abstract

Atopic dermatitis is a T-cell mediated inflammatory skin disease with detected elevated levels of histamine in skin or plasma. In this study, the effects of histamine in a T_H_2 cytokine environment on human keratinocytes and three-dimensional skin models were investigated. These models were used to explore the anti-inflammatory properties of the α-tocopherol-derived long-chain metabolite α-13’-carboxychromanol (α-13’-COOH). Histamine and T_H_2 cytokine-induced proliferation of keratinocytes was studied using a scratch assay. The inflammatory marker interleukin-8 was significantly increased in healthy and T_H_2 cytokine-stimulated keratinocytes and skin models after histamine treatment. The incubation of full-thickness skin models with T_H_2 cytokines and histamine resulted in morphological changes in the epidermal layer, interpreted as hyperkeratosis. α-13’-COOH significantly decreased interleukin-8 in these disease-associated skin models. Histological staining of filaggrin showed skin-strengthening effects following α-13’-COOH treatment, without changes in mRNA expression. Cytokeratin 10 mRNA expression tended to be increased in response to α-13’-COOH. Anti-allergic properties of α-13’-COOH were studied by pre-incubation of human leukocytes with α-13’-COOH. This resulted in reduced sulfido-leukotriene synthesis. The hyperproliferation effect of histamine in atopic dermatitis skin models may be of further interest to the study of disease-associated morphological changes. Moreover, α-13’-COOH is a promising natural compound for the treatment of inflammatory skin diseases.

## 1. Introduction

Atopic dermatitis is the most common chronic pruritic inflammatory skin disease, characterized by dysregulated immune responses. About 15–20% of children and up to 10% of adults in high-income countries suffer from atopic dermatitis [1,2]. Pro-inflammatory cytokines influence the expression of barrier structure proteins in differentiated keratinocytes, which affects natural skin protection. Other factors, such as hyperproliferation of the basal layer resulting in acanthosis or elevated IgE levels are further prominent abnormalities in atopic skin [3,4,5].

Several studies hypothesized that atopic dermatitis in early childhood precedes IgE-mediated type I allergies such as asthma [6,7]. Type I hypersensitivity is characterized by the release of antigen-induced allergy mediators [8]. A prominent mediator is histamine, with high pro-inflammatory activity and the potential to induce pruritus [9]. This mediator is released after degranulation by mast cells and basophils. An elevated mast cell profile was found in the skin of patients with lesional atopic dermatitis skin [10] and significantly higher levels of plasma histamine from atopic dermatitis patients compared to healthy controls [11]. Histamine concentrations from 10 to 1000 µM have been documented in atopic dermatitis skin [12]. Although the pruritus-inducing effect of histamine is well-studied, there are still uncertainties about the pathophysiology of histamine in atopic dermatitis skin and its link to type I allergic reactions.

Investigations of pathophysiological pathways in cell culture models are widely used low-cost tools for disease studies in dermatology. However, single-cell type culture models do not represent the complex structure of the skin. Therefore, disease-associated three-dimensional organotypic skin models are a great tool for the investigation of cell-cell interactions. The role of histamine in three-dimensional skin models has been described, for example, by Gschwandter et al. [13]. Their work showed that the addition of 10 µM histamine to healthy skin models in the early stages of epidermal development strongly reduces keratinocyte differentiation and results in a defective skin barrier. However, this effect could not be detected when histamine was added to fully established skin models.

Here, we investigated the effect of histamine in an atopic dermatitis skin milieu sustained by T_H_2 cytokines (IL-4, IL-13 and IL-31), using human keratinocytes and full-thickness skin models with a developed epidermal layer. Since previous studies suggest that interleukin-8 concentrations in serum and stratum corneum correlate with the severity of atopic dermatitis [14,15,16,17,18], this pro-inflammatory cytokine was used as a biomarker for the evaluation of inflammatory severity in this study.

Several studies investigated dietary supplementation as a treatment option for atopic dermatitis. In a randomized, double-blind, placebo-controlled trial with atopic dermatitis patients, supplementation with a daily dose of 600IU all-rac-α-tocopherol for 60 days showed significant improvement by SCORing Atopic Dermatitis evaluation [19]. Moreover, it has been shown that supplementation with a daily intake of 6·2 mg vitamin E has adverse effects on IgE serum concentrations [20].

A promising natural bioactive compound is the α-tocopherol-derived long-chain metabolite α-13′-carboxychromanol (α-13’-COOH). Previous studies demonstrated that α-13’-COOH occurs in human plasma and is systemically bioavailable [21]. Therapeutic applications were found, including anti-oxidative and anti-inflammatory effects [22,23]. The anti-inflammatory property of α-13’-COOH is based on the inhibition of 5-lipoxygenase (5-LOX) or cyclooxygenase-2. 5-lipoxygenase is a key enzyme for pro-inflammatory sulfido-leukotriene biosynthesis [24,25,26]. Sulfido-leukotrienes are relevant inflammatory mediators in type I allergies. Hence, we hypothesized that α-13’-COOH has, besides its known anti-inflammatory properties, additional anti-allergic effects and is therefore a promising drug in the treatment of inflammatory skin diseases, such as atopic dermatitis. In line with this hypothesis, α-13’-COOH was tested for its anti-allergic potential using clinical test systems. Moreover, anti-inflammatory and therapeutical effects were investigated in our established atopic dermatitis full-thickness skin models.

## 2. Results

### 2.1. The Role of Histamine in an Atopic Dermatitis Cytokine Milieu

#### 2.1.1. Histamine Enhances Proliferation in Healthy and Atopic Dermatitis Keratinocytes

**Figure 1:**
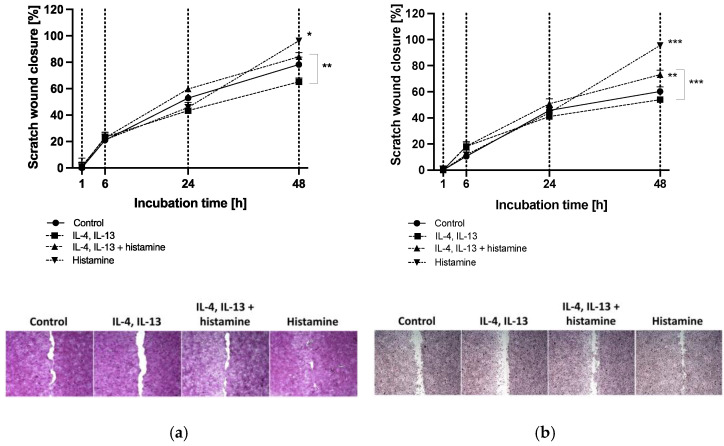
(**a**) HaCaT keratinocyte and (**b**) primary keratinocyte scratch wound closure after 1, 6, 24 and 48 h of incubation. Keratinocytes were either stimulated with T_H_2 cytokines (50 ng/mL IL-4, 50 ng/mL IL-13) with or without 10 µM histamine or cultivated under normal medium conditions with 10 µM histamine. The control group was cultivated without any stimulation. Wound closure is displayed as scratch closure and is presented as mean ± standard error of the mean (SEM) in [%]. Two independent experiments were performed as replicates. Scratch assays were evaluated using six images per sample. Asterisks [*] indicate significant deviations from the control at the respective time point (* *p* < 0.05, ** *p* < 0.01 and *** *p* < 0.001). Cells were stained with haematoxylin-eosin. Images show scratch wound closure of (**a**) HaCaT keratinocytes and (**b**) primary keratinocytes after 48 h of incubation.

Human HaCaT keratinocytes (Figure 1a) and primary human keratinocytes (Figure 1b) were studied as models for epithelial wound closure over an incubation period of 48 h.

The addition of histamine to the culture medium significantly increased the proliferation and migration of HaCaT and primary keratinocytes compared to controls and reached almost 100% after 48 h (*p* < 0.05, *p* < 0.001; Figure 1a,b). Cells under atopic dermatitis conditions (IL-4 and IL-13) showed decreased decline in scratch width in HaCaTs (65.2 ± 2.9%; Figure 1a) and primary keratinocytes (54.0 ± 1.8%; Figure 1b) compared to controls after 48 h. Interestingly, the addition of histamine to the atopic dermatitis milieu enhanced wound healing significantly in primary keratinocytes compared to healthy control after 48 h (73.1 ± 3.5%, *p* < 0.01; Figure 1b) and compared to cells incubated with T_H_2 cytokines after 48 h in both cell types (*p* < 0.01, *p* < 0.001; Figure 1a,b).

**Figure 2:**
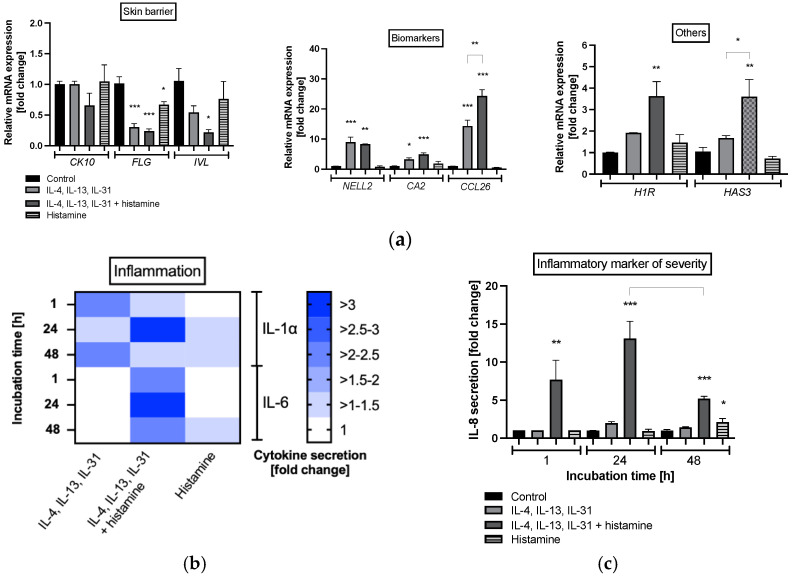
(**a**) Gene expression profiles of primary keratinocytes after 48 h of incubation and (**b**) interleukin (IL) IL-1α, IL-6, (**c**) IL-8 protein expression at after 1, 24 and 48 h of incubation. Keratinocytes were stimulated with T_H_2 cytokines (50 ng/mL IL-4, 50 ng/mL IL-13, 25 ng/mL IL-31) either with or without 10 µM histamine or under normal medium conditions with 10 µM histamine in the absence of T_H_2 cytokines. The control group was cultivated under normal medium conditions without any stimulation. (**a**) Transcript levels are given as normalized relative mRNA expression compared to the untreated control. (**b**,**c**) Cytokine levels are given as fold changes compared to the untreated control at the respective time point. (**a**,**c**) All data are presented as mean ± SEM [fold change]. (**b**) All data in the heatmap are presented as mean [fold change]. Two independent experiments were performed as replicates. Asterisks [*] indicate significant deviations from the untreated control (* *p* < 0.05, ** *p* < 0.01 and *** *p* < 0.001).

In primary keratinocytes, atopic dermatitis cytokines (IL-4, IL-13 and IL-31) significantly decreased mRNA expression of the barrier structure protein filaggrin (*FLG*) without histamine (0.3 ± 0.06-fold, *p* < 0.001; Figure 2a) and in combination with histamine (0.24 ± 0.04-fold; *p* < 0.001; Figure 2a). This effect was also detected for the structure protein involucrin (*IVL*) when the cytokines were combined with histamine (0.2 ± 0.05-fold, *p* < 0.05; Figure 2a), while cytokeratin 10 (*CK10*) remained significantly unchanged. Histamine also reduced *FLG* expression in primary keratinocytes without cytokines (0.7 ± 0.05-fold, *p* < 0.05; Figure 2a).

A significant increase in mRNA expression was measured for atopic dermatitis-associated specific biomarkers such as neural EGFL-like 2 (*NELL2*), carbonic anhydrase 2 (*CA2*), and eotaxin-3 (*CCL26*) following co-incubation of cytokines and histamine (*p* < 0.05, *p* < 0.01, *p* < 0.001, respectively; Figure 2a), while treatment with histamine alone did not change the induction of *NELL2*, *CA2* and *CCL26* significantly. The addition of histamine to the atopic dermatitis milieu enhanced mRNA expression of *CCL26* significantly compared to the T_H_2 cytokine milieu without histamine (*p* < 0.01; Figure 2a). Moreover, histamine increased histamine 1 receptor (*H1R*; 3.6 ± 0.7-fold, *p* < 0.01; Figure 2a) and hyaluronic acid synthase 3 (*HAS3*; 3.6 ± 0.8-fold, *p* < 0.01; Figure 2a) expression under atopic dermatitis conditions compared to healthy controls. Both mRNA expressions were also slightly elevated due to T_H_2 cytokine stimulation without histamine (Figure 2a). mRNA expression of *H4R* was not expressed. *HAS3* mRNA expression was significantly (*p* < 0.05; Figure 2a) and *H1R* mRNA expression tended to be (Figure 2a) elevated after histamine stimulation under atopic dermatitis conditions compared to T_H_2 cytokine stimulation without histamine. Stimulation of keratinocytes cultivated with histamine under normal medium conditions did not markedly alter gene expressions compared to healthy control (Figure 2a).

A pro-inflammatory effect was found in primary keratinocytes when histamine was added in combination with T_H_2 immune cell cytokines to culture medium (IL-4, IL-13 and IL-31). Elevated levels of the pro-inflammatory cytokine IL-1α were detected in atopic dermatitis cytokine milieus with or without histamine after 1, 24 and 48 h compared to healthy control (Figure 2b). Increased IL-6 protein levels were measured only when cells were incubated with the combination of T_H_2 cytokines and histamine at all time points (Figure 2b). The highest levels of both pro-inflammatory cytokines were found when cells were treated with T_H_2 cytokines together with histamine after 24 h (>3-fold; Figure 2b). Treatment with histamine alone did slightly enhance IL-1α secretion compared to control after 24 and 48 h and IL-6 secretion after 48 h (Figure 2b). Moreover, a markedly significant increase in the inflammatory marker of atopic dermatitis severity, namely IL-8, was found after 1 h (7.7 ± 2.6-fold, *p* < 0.01; Figure 2c) 24 h (13.1 ± 2.3-fold, *p* < 0.001; Figure 2c) and 48 h (5.2 ± 0.3-fold, *p* < 0.001; Figure 2c) of incubation with the combination of T_H_2 cytokines and histamine. IL-8 protein decreased significantly after 48 h compared to the protein level measured after 24 h (*p* < 0.05; Figure 2c). In common with IL-1α and IL-6, the highest amount of IL-8 was found after 24 h of incubation. Treatment with histamine alone did enhance secretion of IL-8 compared to control after 48 h (*p* < 0.05; Figure 2c).

#### 2.1.2. Histamine Induces Hyperproliferation in Atopic Dermatitis Skin Models and Enhances Inflammation

**Figure 3:**
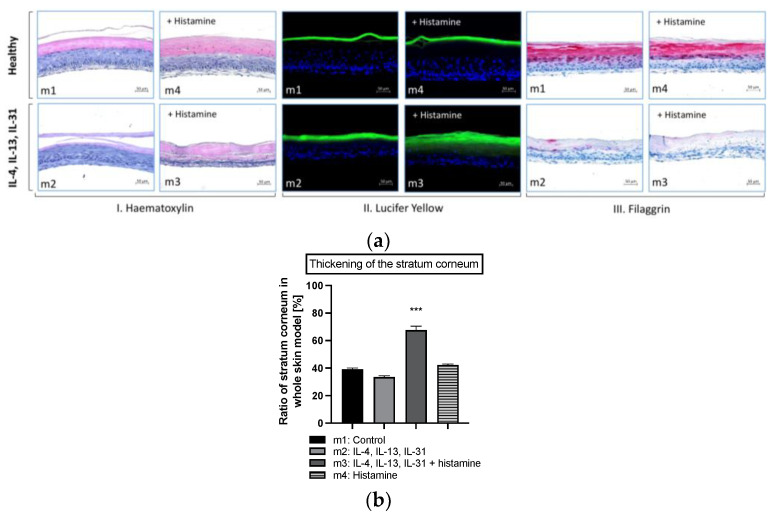
(**a**) Histological evaluation of the skin morphology (I. Hematoxylin), skin permeability effects (II. Lucifer Yellow), skin barrier effects (III. Filaggrin) and (**b**) area ratio of stratum corneum of healthy and atopic dermatitis full-thickness skin models after 12 days of cultivation to air surface (airlift). Atopic dermatitis skin models were stimulated with T_H_2 cytokines (50 ng/mL IL-4, 50 ng/mL IL-13 and 25 ng/mL IL-31) at day 0, 2, 5, 7 and 9 (m2, m3). Healthy skin model m4 and atopic dermatitis skin model m3 were stimulated with 10 µM histamine at day 9. Healthy skin models were cultivated under normal medium conditions (m1, m4). (**a**) Skin models for evaluation of skin morphology were stained with haematoxylin/eosin (I), for evaluation of skin permeability with Lucifer Yellow dye (II) and evaluation of skin barrier effects with filaggrin (III). Scale bar: 50 µm. (**b**) The ratio of the stratum corneum in whole skin models was calculated as the area of the stratum corneum compared to the area of the whole skin model and is given as mean ± SEM in [%] using the image processing program ImageJ (Scale bar: 100 µm). Histological analyses were conducted on three skin models from two independent experiments with four images per skin model. Asterisks [*] indicate significant deviations from the untreated control (*** *p* < 0.001).

Human full-thickness models, consisting of a fibroblast-derived matrix and a fully differentiated epidermis, were used for further investigations on the effect of histamine on atopic skin. Treatment of skin model m2 with atopic dermatitis-relevant cytokines (IL-4, IL-13 and IL-31) during the airlift phase decreased the formation of stratum corneum slightly compared to healthy control m1 (Figure 3a(I),b). However, when histamine was added to the disease-associated skin model (m3) at day 9, thickening of the stratum corneum was morphologically noted on haematoxylin-eosin-stained histological slides compared to all other skin models (Figure 3a(I)). Histological slides showed blue-stained lower layers of the epidermis with nucleated cells in contrast to the pinkish-stained flattened cells with no nuclei of the stratum corneum in the upper layer (Figure 3a(I)). This thickening effect of the stratum corneum in skin model m3 (67.7 ± 2.9%; Figure 3b) compared to healthy skin model m1 (39.2 ± 1.0%; Figure 3b) was confirmed to a significant level (*p* < 0.001; Figure 3b). The addition of histamine to the healthy skin model (m4) at day 9, did not show any morphological changes compared to healthy control m1 (Figure 3a(I); 42.2 ± 0.7%; Figure 3b). 

The skin permeability of the four types of skin models was investigated using the fluorescent dye Lucifer Yellow (Figure 3a(II)). Penetration of the hydrophilic dye into the horny layers of the skin is facilitated when the skin barrier is disrupted. Penetration of Lucifer Yellow into lower epidermal layers was enhanced in hyperproliferated skin models treated with atopic dermatitis-relevant cytokines alone (m2) and with histamine (m3). In contrast, the dye formed a dense fluorescence line located at the top of the horny layer in the skin models m1 and m4, indicating an intact skin barrier (Figure 3a(II)).

Since disturbances in skin structure are strongly related to defects of skin barrier proteins, immunohistochemical staining was conducted. The structural protein filaggrin is localized in the upper layers of the differentiated epidermis and is known to play an important role in the pathogenesis of atopic dermatitis skin [27]. Filaggrin is synthesized in the stratum granulosum from the precursor pro-filaggrin, a main constituent of the keratohyalin granules. Immunohistochemical staining confirmed the presence of filaggrin in the stratum corneum and the upper layers of the stratum granulosum in the healthy skin model m1 (Figure 3a(III)) while lacking in both layers in the disease-associated skin models m2 and m3 (Figure 3a(III)).

**Figure 4:**
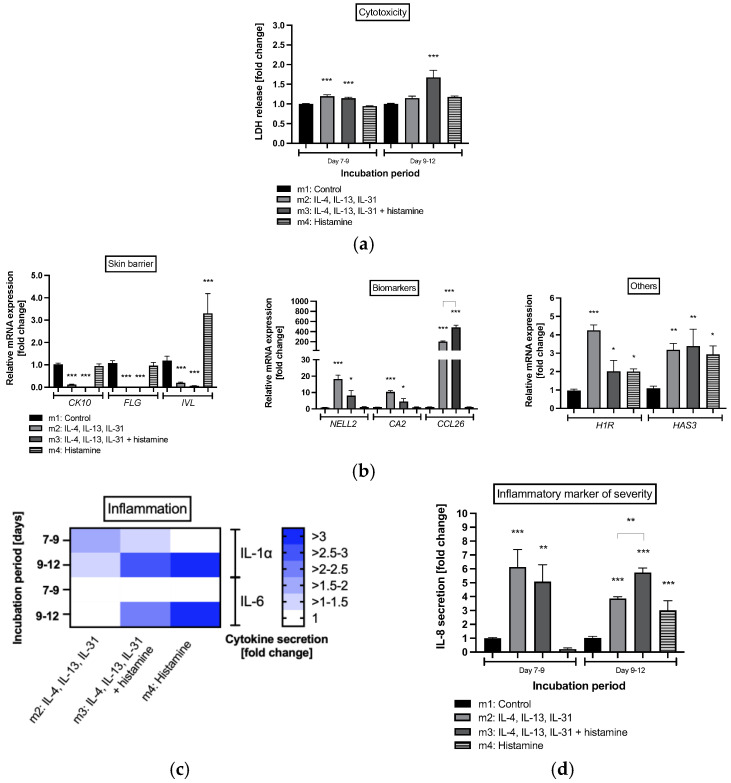
(**a**) Cell viability, (**b**) gene expression profiles from healthy and atopic dermatitis full-thickness skin models after 12 days of cultivation to air surface (airlift), and (**c**) interleukin (IL)-1α, IL-6 and (**d**) IL-8 protein expression after days 7 to 9 and days 9 to 12 of cultivation to air surface (airlift). Atopic dermatitis skin models were stimulated with T_H_2 cytokines (50 ng/mL IL-4, 50 ng/mL IL-13, and 25 ng/mL IL-31) at days 0, 2, 5, 7 and 9 (m2, m3). Healthy skin model m4 and atopic dermatitis skin model m3 were stimulated with 10 µM histamine at day 9. Healthy skin models were cultivated under normal medium conditions (m1, m4). (**a**,**c**,**d**) All data are given as fold changes compared to the untreated control at the respective time points, after day 9 (incubation period days 7 to 9) and day 12 (incubation period days 9 to 12) of incubation. (**b**) Transcript levels are given as relative mRNA expression referred to the untreated control [fold changes]. (**a**,**b**,**d**) All data are presented as mean ± SEM. (**c**) All data in the heatmap are presented as mean. Two independent experiments were performed as replicates. Asterisks [*] indicate significant deviations from the control at the respective time point (* *p* < 0.05, ** *p* < 0.01 and *** *p* < 0.001).

Investigations on cell cytotoxicity showed that histamine not only induced proliferation in keratinocytes from epidermal layers of atopic dermatitis skin model m3 but also significantly enhanced lactate dehydrogenase (LDH) secretion into medium during days 9 to 12 compared to healthy control m1 (1.7 ± 0.2-fold, *p* < 0.001; Figure 4a). Elevated levels of LDH were also detected in disease-associated skin models m2 (1.2 ± 0.04-fold, *p* < 0.001; Figure 4a) and m3 (1.1 ± 0.03-fold, *p* < 0.001; Figure 4a) after days 7–9. Significant levels were no longer detected for atopic dermatitis skin model m2 after days 9–12 (Figure 4a). In the healthy skin model treated with histamine alone (m4), LDH release was not enhanced at any time point (Figure 4a).

Transcript levels of the skin barrier proteins *CK10*, *FLG* and *IVL* were significantly reduced in atopic dermatitis skin models m2 and m3 (*p* < 0.001; Figure 4b). Interestingly, mRNA expression of *IVL* was significantly elevated in response to histamine treatment in healthy skin model m4 (3.3 ± 0.9-fold, *p* < 0.001, Figure 4b). In accordance with the results of the mRNA expression obtained for primary keratinocytes (Figure 2a), atopic dermatitis biomarkers such as *NELL2*, *CA2* and *CCL26* were significantly elevated in skin models m2 and m3 stimulated with T_H_2 cytokines (*p* < 0.05, *p* < 0.001, respectively; Figure 4b). *CCL26* mRNA expression was also significantly elevated when atopic dermatitis skin models were stimulated with histamine (m3) compared to the disease-associated skin model m2 without histamine stimulation (*p* < 0.001; Figure 4b). The transcript levels of *H1R* were also increased for the atopic dermatitis skin models without (m2: 4.2 ± 0.3-fold, *p* < 0.001; Figure 4b) and with (m3: 2.0 ± 0.6-fold, *p* < 0.05; Figure 4b) histamine stimulation as well for *HAS3* (m2: 3.2 ± 0.4-fold, *p* < 0.01; m3: 3.4 ± 0.9-fold, *p* < 0.01, Figure 4b). In the healthy skin model m4, histamine also altered mRNA expression of *H1R* (2.0 ± 0.1-fold, *p* < 0.05; Figure 4b) and *HAS3* (3.4 ± 0.9-fold, *p* < 0.05; Figure 4b) mRNA compared to control but did not affect mRNA expression of biomarkers or skin barrier proteins, except for *IVL*.

A pro-inflammatory effect was observed when T_H_2 cytokines, either with or without histamine, were added to the skin models (Figure 4c,d). Elevated levels of the precursor pro-inflammatory cytokine IL-1α were detected in atopic dermatitis skin models m2 and m3 after days 7 to 9 and days 9 to 12 compared to healthy controls (Figure 4c). The addition of histamine at day 9 induced secretion of IL-1α in healthy skin model m4 and promoted IL-1α levels in disease-associated skin model m3 compared to days 7 to 9. Elevated levels of IL-6 compared to healthy controls were only measured when histamine was added at day 9 to atopic dermatitis skin model m3 and healthy skin model m4 (Figure 4c). The highest levels of both pro-inflammatory cytokines were found when healthy skin model m4 was treated with histamine (>3-fold; Figure 4c). Moreover, a markedly significant increase in the inflammatory marker of atopic dermatitis severity, IL-8, was found in atopic dermatitis skin models m2 (6.1 ± 1.3-fold, *p* < 0.001; Figure 4c) and m3 (5.1 ± 1.2-fold, *p* < 0.01; Figure 4c) during days 7 to 9 and days 9 to 12 (m2: 3.9 ± 0.1-fold, *p* < 0.001; m3: 5.7 ± 0.3-fold, *p* < 0.001; Figure 4c) compared to healthy controls m1. Histamine treatment of the healthy skin model m4 also revealed pro-inflammatory effects after day 12 compared to healthy control by elevated IL-8 levels (3.0 ± 0.7-fold, *p* < 0.001; Figure 4c). This enhancing pro-inflammatory effect of histamine was also found when histamine was added to the atopic dermatitis skin model m3 compared to the untreated disease skin model m2 (*p* < 0.01; Figure 4c). 

### 2.2. Anti-Allergic and Anti-Inflammatory Effects of α-13’-COOH

#### 2.2.1. α-13′-COOH Mediates Anti-Allergic Effects in Leukocytes

**Figure 5:**
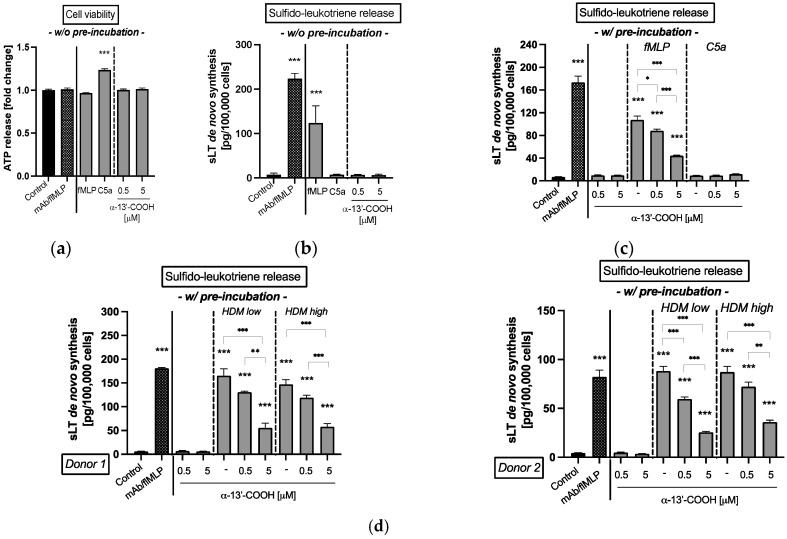
(**a**) Cell viability and (**b**–**d**) de novo synthesis of sulfido-leukotrienes (sLT) from isolated blood leukocytes. (**a**,**b**) Leukocytes were simultaneously incubated without any pre-incubation (w/o pre-incubation) with either fMLP, C5a and 0.5 or 5 µM α-13’-COOH for 50 min. (**c**,**d**) Leukocytes were pre-incubated with (w/ pre-incubation) 0.5 or 5 µM α-13’-COOH or buffer (untreated) for 50 min. Leukocytes used as control were incubated with buffer only. Incubation with an anti-IgE Receptor mAb/fMLP solution is shown as positive control (mAb/fMLP). (**c**) A second unspecific stimulation was conducted either with fMLP (5 µM), C5a (10 nM), or buffer for a further 50 min. (**d**) A second specific stimulation was conducted with an extract from house dust mite (HDM) for specific IgE-mediated response for a further 50 min. Leukocytes from donor 1 and donor 2 were confirmed with a specific IgE level sensitized against HDM extract. HDM extract was applied at two concentrations (HDM low: 2 ng; HDM high: 20 ng). (**a**) Cell viability is presented as the amount of ATP release given as fold change of the control. (**b**–**d**) De novo synthesis of sLT is given as absolute amounts per defined cell number. (**a**–**d**) All data are presented as mean ± SEM. Two independent experiments were performed as replicates. Asterisks [*] indicate significant deviations from the untreated control (* *p* < 0.05, ** *p* < 0.01 and *** *p* < 0.001).

The cell antigen stimulation test (CAST) is used in routine allergy diagnostics to confirm IgE-mediated sensitizations to allergens by quantitative determination of the de novo synthesis of sulfido-leukoptrienes (sLT), which is a group of allergy mediators released by leukocytes. First, the impact of 0.5 or 5 µM α-13’-COOH on human leukocyte viability was investigated. Neither α-13′-COOH nor the control, fMLP or C5a, had a negative impact on the viability of white blood cells at the concentrations used (Figure 5a). Next, we investigated the effect of α-13′-COOH on the release of allergy mediators in leukocytes. In comparison to the positive control (mAb/fMLP) and the unspecific cell activator fMLP (*p* < 0.001, *p* < 0.001; Figure 5b), α-13′-COOH had no impact on the de novo production of sLT (Figure 5b). Stimulation with C5a did not result in any unspecific reactions of the leukocytes by activation of the complement system (Figure 5b).

Data from the literature indicate that a pre-incubation of cells with α-13’-COOH leads to anti-inflammatory effects [28,29]. Hence, this experimental procedure was used to study anti-allergic effects in human leukocytes. The experiments revealed that a pre-incubation of the leukocytes with α-13’-COOH followed by incubation with the unspecific cell activator fMLP dose-dependently reduced de novo synthesis of sLT significantly compared to untreated cells, which were pre-incubated only with buffer (*p* < 0.05, *p* < 0.001, respectively; Figure 5c). A 10-fold higher α-13’-COOH concentration decreased sLT production to more than 50% (0.5 µM α-13’-COOH: 88.1 ± 3.2 pg/100,000 cells; 5 µM α-13’-COOH 44.3 ± 1.0 pg/100,000 cells). Stimulation with C5a had no effect.

Based on these findings, the impact of α-13’-COOH was tested in primary human leukocytes isolated from blood. Two blood donors with known sensitization to the HDM extract were used. Pre-incubation of leukocytes with 0.5 or 5 µM α-13’-COOH significantly reduced de novo production of sLT after stimulation with HDM extract compared to the untreated control (*p* < 0.01, *p* < 0.001, respectively; Figure 5d). A 10-fold higher α-13’-COOH concentration decreased sLT production to more than 50% in both donors, which is comparable to the effect size after fMLP stimulation (Figure 5c).

**Figure 6:**
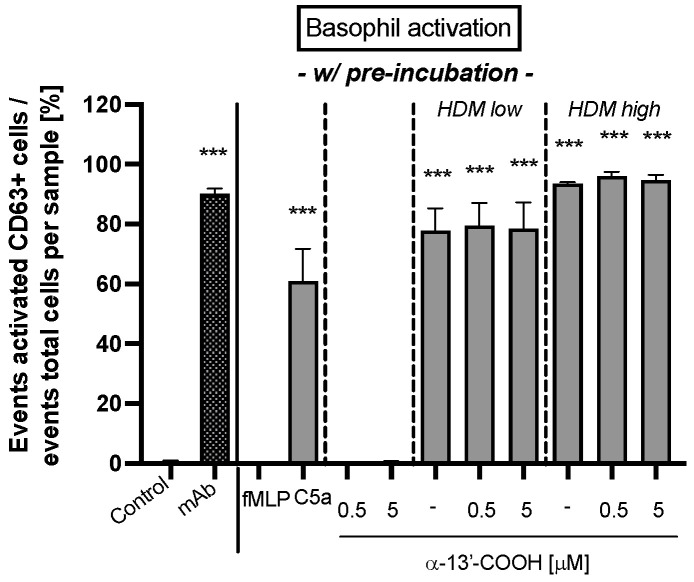
Basophil activation in whole blood from donors with confirmed specific IgE-sensitized immune cells against house dust mite (HDM) extract. Whole blood cells were pre-incubated with 0.5 or 5 µM α-13’-COOH or buffer (untreated control) for 50 min. Blood cells used as controls were pre-incubated with buffer. A second unspecific basophil activation was tested either with fMLP (5 µM), C5a (10 nM), or buffer. Activation of basophils with HDM extract was investigated as specific IgE-mediated response. The HDM extract was applied in two concentrations (HDM low: 2 ng; HDM high: 20 ng). A specific anti-IgE receptor mAb was used as positive control (mAb). Activated basophils were stained with a mixture of monoclonal antibodies against human cluster of differentiation (CD) 63 labeled with fluorescein isothiocyanate (anti-CD63-FITC) and against human C-C chemokine receptor type 3 (CCR3) labeled with phycoerythrin (anti-CCR3-PE). Basophil activation is given as the event of activated CD63-positive basophils in relation to the absolute events of each sample in percent. All data are presented as mean ± SEM. Two independent experiments were performed as replicates. Asterisks [*] indicate significant deviations from the untreated control (*** *p* < 0.001).

Finally, the effect on basophil activation after pre-incubation with α-13’-COOH was analyzed. The study showed that fMLP, the anti-FcεRI mAb, and the HDM extract activated the CD63 signaling cascade significantly (*p* < 0.001; Figure 6), whereas C5a and α-13’-COOH did not. Moreover, α-13’-COOH did not alter basophil activation after HDM extract stimulation compared to the untreated control.

#### 2.2.2. α-13′-COOH Affects Skin Barrier Integrity in Atopic Dermatitis Skin Models and Reduces Inflammation

**Figure 7:**
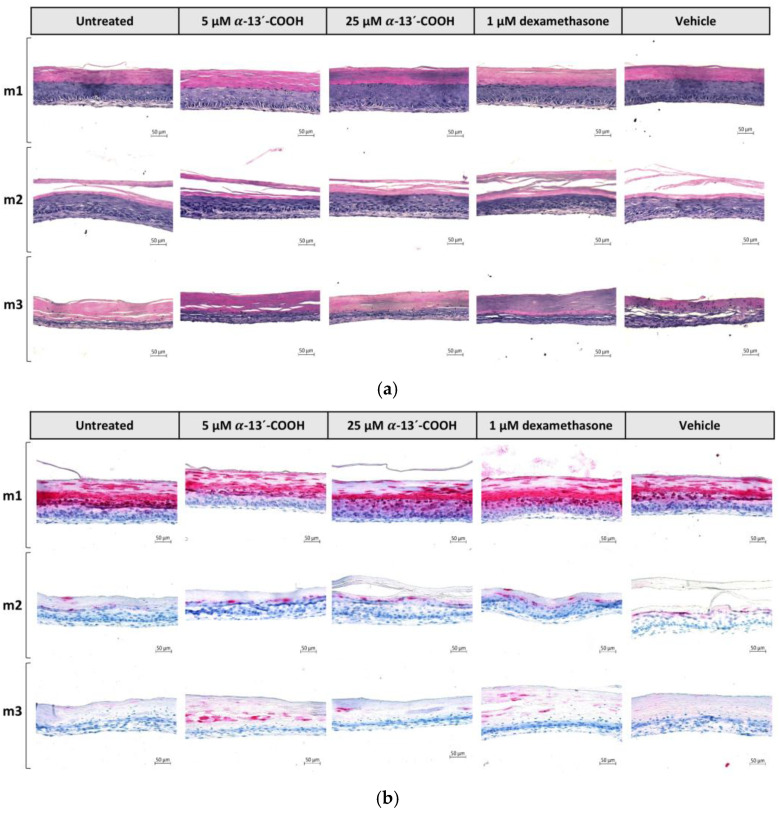

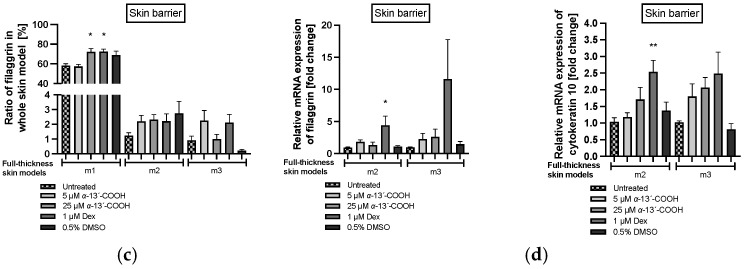
(**a**,**b**) Histological, (**c**) quantitative evaluation, and (**d**) gene expression profile of filaggrin in healthy and atopic dermatitis full-thickness skin models after 12 days of cultivation to air surface (airlift). Healthy skin models were cultivated under normal medium conditions (m1). Atopic dermatitis skin models were stimulated with T_H_2 cytokines (50 ng/mL IL-4, 50 ng/mL IL-13 and 25 ng/mL IL-31) at days 0, 2, 5, 7 and 9 (m2, m3). Atopic dermatitis skin model m3 was additionally stimulated with 10 µM histamine at day 9. Skin models m1, m2 and m3 were pre-incubated with either 5 µM α-13’-COOH, 25 µM α-13’-COOH, 1 µM dexamethasone (Dex; positive control), or 0.5% DMSO (vehicle control) at days 7 and 9, or were left untreated as disease control (untreated). (**a**) Skin models for evaluation of skin morphology were stained with haematoxylin-eosin and (**b**) for evaluation of skin barrier effects with filaggrin. Scale bar: 50 µm. (**c**) Ratio of filaggrin expression in whole skin models is given as the area of filaggrin in whole skin models in relation to the area of the whole skin model in [%] using ImageJ software (Scale bar: 100 µm). (**d**) Transcript levels are given as relative mRNA expression referred to the untreated disease control [fold change]. (**c**,**d**) All data are presented as mean ± SEM. Two independent experiments were performed as replicates. Histological analyses were conducted on three skin models from two independent experiments with four images per skin model. Asterisks [*] indicate significant deviations from the untreated disease control (* *p* < 0.05 and ** *p* < 0.01).

The anti-inflammatory properties of α-13′-COOH have been previously described [24,25,26]. In addition, our studies revealed an anti-allergic effect through intervention with the de novo synthesis of sLT (Figure 5). Therefore, the α-tocopherol-derived metabolite α-13’-COOH was tested for its therapeutic properties on the healthy skin model m1 and the atopic dermatitis skin models m2 and m3. All three skin models were treated with either 0.5 or 5 µM α-13’-COOH, 1 µM dexamethasone (positive control), or 0.5 % DMSO (vehicle control) at days 7 and 9. The untreated controls of each skin model type did not receive any treatment. Skin models were harvested at day 12. None of the compounds induced visual abnormalities in skin structure compared to the respective skin model controls (Figure 7a). 

While prior investigations demonstrated that the atopic dermatitis skin models show skin barrier defects due to the loss of filaggrin (Figure 3a(I)), a pre-incubation with 5 µM α-13’-COOH increased filaggrin content in atopic dermatitis skin model m3 compared to the untreated disease controls in a manner comparable to dexamethasone (Figure 7c). The filaggrin-elevating effect of α-13’-COOH was not observed at the transcription level, while 1 µM dexamethasone increased *FLG* mRNA expression in skin models m2 (4.4 ± 1.4-fold, *p* < 0.05; Figure 7d) and m3 (11.6 ± 6.2-fold, Figure 7d). However, the mRNA levels of *CK10* have been increased in tendency, following treatment with 25 µM α-13’-COOH in both disease-associated skin models m2 (1.7 ± 0.4-fold; Figure 7d) and m3 (2.1 ± 0.3-fold; Figure 7d). *CK10* is another important protein for skin integrity. Dexamethasone also induced mRNA expression of *CK10* to a significant level in both models (m2: 2.5 ± 0.3-fold, *p* < 0.01; m3: 2.5 ± 0.6-fold; Figure 7d).

**Figure 8:**
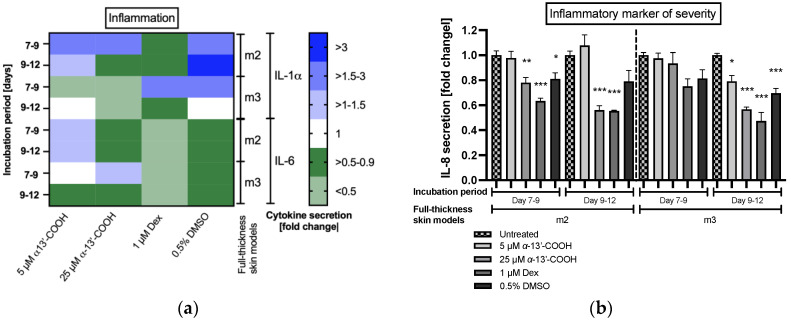
(**a**) Interleukin (IL)-1α and IL-6 protein expression and (**b**) IL-8 protein expression in atopic dermatitis full-thickness skin models after days 7 to 9 and days 9 to 12 of cultivation to air surface (airlift). Atopic dermatitis skin models were stimulated with T_H_2 cytokines (50 ng/mL IL-4, 50 ng/mL IL-13 and 25 ng/mL IL-31) at days 0, 2, 5, 7 and 9 (m2, m3). Atopic dermatitis skin model m3 was additionally stimulated with 10 µM histamine at day 9. Skin models m2 and m3 were pre-incubated with either 5 µM α-13’-COOH, 25 µM α-13’-COOH, 1 µM dexamethasone (Dex; positive control), or 0.5% DMSO (vehicle control) at days 7 and 9, or were left untreated as disease control (untreated). Cytokine levels are given as fold change to untreated disease control at the respective time points, after day 9 (incubation period days 7 to 9) and day 12 (incubation period days 9 to 12) of incubation. **(a)** All data in the heatmap are presented as means. (**b**) All data are presented as means ± SEM. Two independent experiments were performed as replicates. Asterisks [*] indicate significant deviations from the untreated disease control at the respective time point. (* *p* < 0.05, ** *p* < 0.01 and *** *p* < 0.001).

An anti-inflammatory effect of α-13’-COOH was detected by evaluating the cytokine secretion (Figure 8). Our previous results showed that histamine increases the secretion of the pro-inflammatory cytokines IL-1α, IL-6 and IL-8 in our atopic dermatitis skin models m2 and m3, when added at day 9 to the medium (Figure 4c,d). Anti-inflammatory properties of 25 µM α-13’-COOH were detected as reflected in lower levels of IL-1α and IL-6 secretion in both diseases-associated skin models m2 and m3 compared to disease controls (Figure 8a). This effect was comparable to that of dexamethasone. Vehicle control with DMSO showed reducing effects on IL-6 secretion and pro-inflammatory properties for IL-1α (Figure 8a).

Significant anti-inflammatory effects were found for the inflammatory marker of atopic dermatitis severity IL-8. Application of 25 µM α-13’-COOH significantly reduced the secretion of IL-8 in atopic dermatitis skin models m2 (0.6 ± 0.03-fold, *p* < 0.001; Figure 8b) and m3 (0.6 ± 0.02-fold, *p* < 0.001; Figure 8b) at days 9 to 12 compared to the disease control, similar to dexamethasone (m2: 0.6 ± 0.03-fold, *p* < 0.001; m3: 0.5 ± 0.07-fold, *p* < 0.001; Figure 8b). Secretion of IL-8 was also significantly reduced after days 7 to 9 in atopic dermatitis skin model m2 by 25 µM α-13’-COOH (0.8 ± 0.04-fold, *p* < 0.01; Figure 8b). Vehicle control with DMSO showed reducing effects after the incubation period days 7 to 9 in skin model m2 (0.8 ± 0.05-fold, *p* < 0.05; Figure 8b) and in skin model m3 after day 12 (0.7 ± 0.04-fold, *p* < 0.001; Figure 8b). 

## 3. Discussion

The pathogenesis of atopic dermatitis is complex and its link to IgE-mediated type I allergies has not yet been clearly elucidated. It is still controversially discussed whether systemic inflammation triggers barrier disruption (the inside-out hypothesis) or skin barrier defects encourage immunologic reactions (the outside-in hypothesis) [4]. Nevertheless, both explanatory models support the hypothesis that a defective skin barrier facilitates the entry of antigens, resulting in an allergic reaction and the release of histamine by immune cells. Skin-barrier defects in skin models were generated in our study by simultaneous treatment with the T_H_2 cytokines IL-4, IL-13 and IL-31, resulting in the downregulation of skin barrier proteins, such as *CK10*, *FLG* and *IVL*. This is congruent with other atopic dermatitis skin models described previously [30,31,32]. Furthermore, the upregulation of disease-associated biomarkers, such as *NELL2*, *CA2* and *CCL26*, indicates the prevalence of an immune response in the atopic dermatitis skin model. The chemokine *CCL26* promotes infiltration of eosinophils and is upregulated by IL-4 via the Janus kinase (JAK) 1/2-signal transducer and activator of the transcription (STAT) 6 pathway in keratinocytes [33]. The pathophysiologic role of *NELL2* and *CA2* is not clearly understood, although their gene expression is induced in the epidermis of human skin equivalents cultured with IL-4 and IL-13 [34].

Furthermore, an upregulation of *HAS3* mRNA expression in a T_H_2 cytokine milieu was found in this study. Whereas *HAS3* was only upregulated after stimulation with the combination of T_H_2 cytokines plus histamine in primary keratinocytes, its mRNA expression was increased in all skin models m2, m3 and m4 compared to the healthy control model m1. The metabolic function of hyaluronan synthases (HAS) is to transfer hyaluronic acid, an essential extracellular polysaccharide produced by keratinocytes, to the extracellular matrix [35]. Hyaluronan plays an important role in repair function and skin homeostasis, however, its epidermal function is not clearly identified. *HAS3* mRNA expression was found to be upregulated in lesional skin biopsies from atopic dermatitis patients compared to healthy or non-lesional skin, which may be seen as an indication of alterations in epidermal matrix metabolism in atopic dermatitis lesional skin [36,37].

In total, three histamine receptors have been described in human keratinocytes, namely *H1R*, *H2R* and *H4R*; although, anti-allergic and anti-pruritic therapy currently targets *H1R* [11,38,39,40]. The binding of histamine to *H1R* mediates hypersensitivity reactions, which promote typical inflammatory skin symptoms, such as redness, edema, or pruritus [41]. In the present study, histamine significantly elevated *H1R* transcript levels in primary keratinocytes cultivated in a T_H_2 cytokine milieu. A significant influence of histamine on *H1R* mRNA levels was also found in the atopic dermatitis skin model m3 and the healthy skin model m4. Furthermore, upregulation of *H1R* mRNA expression at a significant level was measured in skin model m2 cultivated in a T_H_2 cytokine setting without histamine. This is in line with the finding that the *H1R* mRNA level can be upregulated by histamine or IL-4 [42] and findings of the higher expression of *H1R* mRNA in keratinocytes from patients with atopic dermatitis [38]. In addition to the expression of *H1R*, Glatzer et al. [38] demonstrated that the *H4R* receptor was highly expressed in keratinocytes from patients with atopic dermatitis. Stimulation of *H4R* induces the proliferation of keratinocytes and may therefore play a role in epidermal hyperplasia. Since we observed proliferative effects in our keratinocytes while no expression of *H4R* mRNA was detected, we can speculate that keratinocyte proliferation induced by activation of the histamine signaling cascade is complex, involving different triggers and pathways, which still needs further investigation.

Several studies indicate that histamine is a pro-inflammatory mediator [43,44,45,46]. Matsubara et al. [47] demonstrated that stimulation of epidermal keratinocytes with histamine induces the synthesis of pro-inflammatory cytokines IL-6 and IL-8 as a late allergic response via *H1R* by induction of Extracellular-signal Regulated Kinases (ERK) and Nuclear factor-κB (NF-κB) signaling. Recent studies reported that IL-8 is a suitable biomarker for monitoring atopic dermatitis severity and therapeutic effects in the clinical context [14,15,16,18]. Furthermore, increases in IL-8 in serum can be used as a preferable biomarker to identify the status of other atopic diseases, such as asthma [48]. In this study, we demonstrate that histamine increases protein levels of IL-8 in primary keratinocytes and the full-thickness skin models m3 and m4 significantly. Hence, we conclude that histamine is also able to promote inflammatory severity in atopic dermatitis full-thickness skin models.

Epidermal hyperproliferation is a characteristic dermatopathological phenotype in atopic dermatitis. Histamine might strongly contribute to the hyperproliferation of keratinocytes. In the present study, mitogenic effects of histamine were seen in HaCaTs and primary keratinocytes after 48 h. A significant proliferative effect of human foreskin keratinocytes was also described after 48 h incubation with histamine compared to control [38]. Glatzer et al. [38] also note that keratinocytes from different healthy sources showed various responses to histamine. While neonatal keratinocytes stimulated with histamine showed increased proliferation, keratinocytes from hair roots did not. In this publication, it is further stated that keratinocytes derived from atopic dermatitis patients showed increased proliferation in response to histamine. These findings are in line with our findings in the scratch assay and promote the in vitro reproducibility of the role of histamine in an atopic dermatitis skin milieu. However, several other factors are involved in keratinocyte hyperproliferation and the underlying pathophysiological mechanism and the role of histamine remain elusive. Hyperproliferation and abnormal differentiation of keratinocytes lead to epidermal structural changes in the skin, such as hyperkeratosis. Hyperkeratosis refers to an increased thickening of the stratum corneum and occurs markedly in chronic skin lesions of patients with atopic dermatitis [49]. An abnormal proliferation effect of histamine in healthy primary keratinocytes, keratinocytes from atopic dermatitis patients, or healthy skin equivalents, was already published in the literature [13,38,50,51]. The role of T_H_2 cytokines, such as IL-4, on abnormal epidermal proliferation, is discussed controversially [52,53,54,55]. In this study, we show for the first time, that the addition of histamine to disease-associated full-thickness skin models stimulated with T_H_2 cytokines (IL-4, IL-13, IL-31) results in morphological changes of the epidermal layer, interpreted as hyperkeratosis.

Acute lesions of atopic dermatitis skin are characterized by spongiosis, flare-ups, itchiness, edema and infiltration of T_H_2-dominated immune cells. The thickening of the epidermis and hyperkeratosis is more prominent in chronic stages of atopic dermatitis but is also present in acute stages [56]. A recent study pointed out that it is still unclear whether the switch from acute to chronic phase is driven by an altered molecular mechanism or is more likely the result of different grades of the inflammatory response [57]. Previous studies mentioned that a switch from T_H_2 to T_H_1 cell-mediated signaling is mainly involved in the shift from acute to chronic stages [58,59,60]. Fujimoto et al. [61] postulated that histamine may have negative-feedback signaling effects on existing T_H_2-dominated inflammation by supporting the secretion of T_H_1 cell-associated mediators and suppressing T_H_2 cell-related chemokines. Nonetheless, data are scarce as to whether histamine plays a key role as a promoter for the switch from acute to chronic atopic dermatitis stages and its role to induce hyperkeratosis.

We assumed that the anti-inflammatory α-tocopherol-derived long-chain metabolite α-13’-COOH is a promising compound for the treatment of atopic dermatitis or other atopic diseases. As α-13’-COOH inhibits 5-LOX [25,62], we investigated if α-13’-COOH shows also anti-allergic properties in clinical test systems, such as cellular antigen stimulation test (CAST) and basophil activation test (BAT). Our results demonstrated a significant inhibitory effect on the de novo synthesis of sLT when blood leukocytes were pre-incubated with 0.5 or 5 µM α-13’-COOH independent from unspecific (formyl-methionyl-leucyl-phenylalanin; fMLP) and specific (HDM extract) cell stimulation. However, pre-incubation of α-13’-COOH had no marked impact on basophil activation after HDM extract stimulation indicating that α-13’-COOH does not affect receptor-associated activation of the cells but interferes with later steps in sLT production after cell stimulation. Hence, the exact modes of mechanisms of α-13’-COOH still need to be investigated. In general, the release of sLT occurs after antigen-induced activation of IgE-bearing immune cells via the arachidonic acid pathway and may play a role in inflammatory atopic conditions. Soter and colleagues [63] found that intracutaneous injections of LTC4, LTD4 and LTE4 into human skin cause erythema and wheal formation. Clinical therapies targeting the sLT pathway or 5-LOX inhibition are limited, but there have been promising initial results in the treatment of other atopic diseases such as allergic rhinitis and asthma [64]. The clinical relevance of 5-LOX inhibitors is still under controversial discussion [65]. Here, we show for the first time an sLT-reducing effect of α-13’-COOH using CAST.

This is the first report about the influence of α-13’-COOH on filaggrin in T_H_2 cytokine-stimulated skin models. α-13’-COOH increased expression of filaggrin, a key protein for epidermal structure and barrier function, in atopic dermatitis skin model m3 compared to the untreated disease control model; thus, this vitamin-E derivative may have promising strengthening effects on the skin barrier. Since these findings are not occurring at the transcription level, we assume that this effect may not be mediated by elevated gene expression. Transcripts may have higher stability and can be read multiple times or α-13’-COOH increases protein stability. However, elevated levels of mRNA expression of another skin barrier protein, namely *CK10* were detected, which supports a potential skin-strengthening effect of α-13’-COOH.

Noticeable effects were found for the measurement of the inflammatory severity marker IL-8. Incubation of the atopic dermatitis skin models m2 and m3 with 25 µM α-13’-COOH decreased the expression of the inflammatory severity marker IL-8 of atopic dermatitis significantly compared to the respective disease controls. This anti-inflammatory effect was comparable to that of dexamethasone. Anti-inflammatory effects were also discovered after treatment with 25 µM α-13’-COOH for IL-1α and IL-6 secretion.

In general, further studies are necessary to better understand the underlying mechanism of α-13’-COOH as a therapeutic agent for the treatment of atopic diseases and its effect on skin mechanism.

## 4. Materials and Methods

### 4.1. Test Substance

Semi-synthesis of α-13’-carboxychromanol (α-13’-COOH) was performed after the isolation of garcinoic acid from Garcinia kola seeds as previously described [66,67].

### 4.2. Primary Keratinocytes and HaCaT Culture

Human HaCaT cells (kindly provided by Prof. Norbert Fuesing, Heidelberg, Germany) and human primary keratinocytes (generated from juvenil phimosis; 4739-03/16) were cultured in 175 cm^2^ cell culture flasks (Greiner bio-one, Frickenhausen, Germany) as previously described [68]. 

### 4.3. Scratch Wound Assay

HaCaTs and primary keratinocytes were seeded in 12-well plates (Greiner bio-one, Maybachstrasse, Frickenhausen, Germany) with 2×10^5^ cells/mL (2 mL medium per well) and cultured for 48 h to a confluence of 80–90%. HaCaTs were cultured in Dulbecco’s modified Eagle’s medium (Promocell, Sickingenstrasse, Heidelberg, Germany) supplemented with 10% fetal bovine serum (PAN-Biotech, Am Gewerbepark, Aidenbach, Germany) and antibiotic-antimycotic solution (BioConcept, Paradiesrain, Alschwil, Switzerland) at 37 °C in a 5% CO_2_ atmosphere. Human normal epidermal keratinocytes were cultured in a keratinocyte growth medium 2 (Promocell, Sickingenstrasse, Heidelberg, Germany) and 0.5% gentamicin (ThermoFisher, Waltman, MA, USA) at 37 °C and in a 5% CO_2_ atmosphere. Cell monolayers were scratched with a standard 1000 µL sterile pipette tip and washed with warm PBS (Bioconcept, Paradiesrain, Allschil, Switzerland). Then, cells were stimulated with 50 ng/mL IL-4 and 50 ng/mL IL-13 (7Bioscience, Dekan-Martin-Strasse, Neuenburg, Germany) with or without 10 µM histamine (Sigma Aldrich, Frankfurter Strasse, Darmstadt, Germany) in culture medium. Cells cultured in normal culture medium served as untreated control. Cells were incubated for 1, 6, 24 and 48 h at 37 °C in a 5% CO_2_ atmosphere. After incubation, cells were fixed with 4% formalin (Dr. K. Hollborn & Söhne, Brahestrasse, Leipzig, Germany) and stained with haematoxylin and eosin (Merck, Frankfurter Strasse, Darmstadt, Germany). The imaging of scratch closure and evaluation was carried out using a VHX 950F digital microscope (Keyence Deutschland, Siemensstrasse, Neu-Isenburg, Germany).

### 4.4. Cell Culture for Analysis of Gene Expression and Cytokine Secretion

Primary keratinocytes were seeded in 12-well plates (Greiner bio-one, Maybachstrasse, Frickenhausen, Germany) with 5 × 10^4^ cells/mL (2 mL medium per well) to adhere overnight. The following day, cells were washed with fresh medium and 1.88 mM CaCl_2_ solution (Serumwerk Bernburg, Hallesche Landstrasse, Bernburg, Germany) for 48 h. cells were stimulated with 50 ng/mL IL-4, 50 ng/mL IL-13 and 25 ng/mL IL-31 (7Bioscience, Dekan-Martin-Strasse, Neuenburg, Germany) with or without 10 µM histamine (Sigma Aldrich, Frankfurter Strasse, Darmstadt, Germany). Cells cultured in normal medium served as untreated control. For the analysis of cytokine secretion, culture supernatants were collected and replaced after 1, 24 and 48 h. Gene expression analyses were conducted after 48 h.

### 4.5. Cultivation of Full-Thickness Skin Models

Full-thickness skin models were cultured based on previous publications [69,70,71]. Normal human dermal fibroblasts (Promocell, Sickingenstrasse, Heidelberg, Germany) were cultured in Dulbecco’s modified Eagle’s medium (DMEM; BioConcept, Paradiesrain, Alschwil, Switzerland) supplemented with 2% fetal calf serum (FCS; PAN-Biotech, Gewerbepark, Aidenbach, Germany), 0.5% gentamicin (Thermo Fisher, Waltman, MA, USA), 5 μg/mL insulin and 5 ng/mL human fibroblastic growth factor (Cellsystems, Triosdorf, Germany) at 37 °C in a 5% CO_2_ atmosphere to approximately 90% confluence. Fibroblasts were harvested by trypsin-EDTA (Thermo Fisher, Waltman, MA, USA) treatment, seeded into 12-well inserts (Greiner Bio-One, Maybachstrasse, Frickenhausen, Germany) giving a final concentration of 1 × 10^5^ fibroblasts/mL and incubated at 37 °C in a 5% CO_2_ atmosphere. Dermis was developed without a collagen matrix, only by fibroblasts in a submerse medium consisting of DMEM, 10% FCS and 0.5% gentamicin supplemented with 150 μg/mL ascorbic acid (Sigma Aldrich, Frankfurter Strasse, Darmstadt, Germany) for 3 weeks. The medium was changed every 2–3 days. Human normal epidermal keratinocytes were seeded with a density of 2×10^5^ cells/insert on the top of the dermis at day 21. The dermal layer was coated with 50 μg/mL fibronectin (Promocell, Sickingenstrasse, Heidelberg, Germany) 30 min before the seeding of keratinocytes. Skin models were cultured in a submerse medium consisting of keratinocyte basal growth medium 2 (Promocell, Sickingenstrasse, Heidelberg, Germany), 5% FCS, 150 μg/mL ascorbic acid and 0.5% gentamicin for further 7 days. Medium was changed twice, while 1.88 mM CaCl_2_ was added to the last medium change. At day 28 the skin models were placed into 12-well ThinCert™ cell culture plates (Greiner bio-one, Maybachstrasse, Frickenhausen, Germany) and lifted to the medium-air interface (airlift phase) for 12 days. The culture medium was composed of DMEM + Ham’s F12 (ratio 1:1), 5% FCS, 5 µg/mL insulin, 13.51 µg/mL, adenine, 0.5% gentamicin, 0.33 µg/mL hydrocortisone, 3.99 × 10^−9^ mg/mL tri-iodothyronine, 5 µg/mL holo-transferrin, 150 μg/mL ascorbic acid and 1.88 mM CaCl_2_. 

A healthy skin model (m1) was set up without any further stimulation at the beginning of the airlift phase. Atopic dermatitis skin models (m2 and m3) were stimulated with 50 ng/mL IL-4, 50 ng/mL IL-13 and 25 ng/mL IL-31 at days 0, 2, 5, 7 and 9 simultaneously to medium change. The disease-associated skin model 3 (m3) was further stimulated with 10 µM histamine at day 9. In addition, a healthy skin model 4 (m4) was also stimulated with 10 µM histamine at day 9. All four skin models (m1, m2, m3, m4) were harvested for further analysis at day 12 in the airlift phase. 

### 4.6. Treatment of Full-Thickness Skin Models

During cultivation in the airlift phase, 5 and 25 µM α-13’-COOH, 1 µM dexamethasone (Dex), and 0.5% dimethylsulfoxid (DMSO; Sigma Aldrich, Frankfurter Strasse, Darmstadt, Germany) were added to the culture medium of skin models m1, m2 and m3 at day 7 and day 9. Dexamethasone was used as positive control, and 0.5% DMSO served as vehicle control. The control group of each skin model type did not receive any treatment.

### 4.7. Determination of Cell Viability and Cytotoxicity

Cell viability was determined by measuring the adenosine triphosphate (ATP) concentration in viable cells according to manufacturers’ recommendations (PerkinElmer, Waltham, MA, USA) using a luminescence reader (LumiSTAR Galaxy; BMG Labtech, Ortenberg, Germany). ATP concentrations were calculated using an eight-point calibration curve. Cytotoxicity effects were determined by measuring the release of LDH according to manufacturers’ recommendations (Promega, Gutenbergring, Walldrof, Germany) using a microplate reader at 490 nm (POLARstar Galaxy; BMG Labtech, Allmendgrün, Ortenberg, Germany). LDH release was calculated as fold change using the untreated control as a reference set to 1.

### 4.8. Determination of Cytokine Levels

Cytokine release was quantified using human (IL-8 (CXCL8), IL-1α (R&D Systems, Minneapolis, MN, USA) and IL-6 (Mabtech, Nacka Strand, Stockholm, Sweden) enzyme-linked immunosorbent assay (ELISA) kits according to the manufacturers’ protocols using a microplate reader (FLUOstar Galaxy, BMG Labtech, Allmendgrün, Ortenberg, Germany) at 450 nm and 620 nm (reference wavelength). Cytokine concentrations were calculated using a four-parameter curve fit with an eight-point calibration curve.

### 4.9. RNA Isolation, cDNA Synthesis and Quantitative Real-Time PCR (RT-qPCR)

Sample preparation of primary keratinocytes for RNA isolation was conducted after the removal of cell culture supernatants. Human cells were lysed by adding lysis buffer (Qiagen, Hilden, Nordrhein-Westfalen, Germany) containing 10 µL/mL β-mercaptoethanol (Sigma Aldrich, Frankfurter Strasse, Darmstadt, Germany) and incubated on ice for 3 min and a further 3 min under shaking. Lysates were loaded to QIA Shredder spin columns (Qiagen, Hilden, Nordrhein-Westfalen, Germany) and centrifuged (2 min, 4 °C, 10,000× *g*).

Sample preparation of skin models for RNA isolation was conducted by milling the skin models in a lysis buffer (Qiagen, Hilden, Nordrhein-Westfalen, Germany) containing 10 µL/mL β-mercaptoethanol (Sigma Aldrich, Frankfurter Strasse, Darmstadt, Germany) and steel balls for 2 min at 30 Hz (Retsch^®^ MM 301; Retsch GmbH, Retsch-Allee, Haan, Germany). Afterward, tissue was digested by the addition of proteinase K (Applichem, Ottoweg, Darmstadt, Germany) and incubated in a thermomixer (10 min, 55 °C).

RNA purification, reverse transcription and real-time polymerase chain reaction were carried out as previously described [68]. Relative gene expression was calculated using the 2-ΔΔ Ct method using β-actin as a housekeeping gene for normalization [72]. Primers used for gene expression analyses are listed in Table 1 (Eurofins Genomics, Anzinger Strasse, Ebersberg, Germany) and Table 2 (Qiagen, Hilden, Nordrhein-Westfalen, Germany).

### 4.10. Determination of Cellular Antigen Stimulation and Specific Anti-Allergic Effects

The anti-allergic effect of α-13’-COOH was measured using the Cellular Antigen Stimulation Test kit (Bühlmann Laboratories, Schönenbuch, Switzerland) with slight modifications. Blood leucocytes were isolated by the addition of dextran solution. After 30 min, the plasma fraction was separated and centrifugated (10 min, 160× *g*). Cell pellets were resuspended in IL-3-containing buffer to an equal volume to the initial blood volume. The number of leukocytes was measured immediately (Casy cell counter, Schärfe System, Reutlingen, Germany). The buffer was used as a solvent for all compounds. A 5 µM quantity of fMLP (Sigma Aldrich, Frankfurter Strasse, Darmstadt, Germany) was used as an unspecific cell activator and 10 nM recombinant human complement component 5a (C5a; Sigma Aldrich, Frankfurter Strasse, Darmstadt, Germany) was used as complement system activator. A provided stimulation control, a solution of an anti-Fc Epsilon R1 monoclonal antibody (anti-FcεRI mAb) and fMLP, was used as positive control. Three different experimental setups were conducted as follows.

First, α-13’-COOH was diluted with buffer and combined with isolated leukocytes from healthy donors to a concentration of 0.5 and 5 µM in microtiter plates and tubes. fMLP, C5a and the anti-FcεRI mAb/fMLP solution were added separately. Mixtures were incubated (50 min, 37 °C). The microtiter plate was used for ATP quantification. The tubes were vortexed and centrifugated (6 min, 4 °C. 1000× *g*). Cell supernatants were immediately analyzed for de novo synthesis of sulfido-leukotrienes (LTC4, LTD4, LTE4) in pre-coated ELISA plates using a microplate reader at 405 nm (FLUOStar Galaxy; BMG Labtech, Allmendgrün, Ortenberg, Germany).

In the second experimental setup, leukocytes were pre-incubated with or without 0.5 and 5 µM α-13’-COOH for 50 min (37 °C). Afterward, fMLP, C5a, positive control, or buffer were added to the tubes and incubated for a further 50 min at 37 °C, followed by vortexing and centrifugation (6 min, 4 °C, 1000× *g*). sLT concentrations were quantified as described in the first experiment.

Finally, specific allergic reactions were studied. For this, leukocytes from two blood donors with a confirmed type I allergy against HDM extract from *Dermatophagoides Pteronyssinus* (Phadia, Munzinger Strasse, Freiburg, Germany) were pre-incubated with 0.5 and 5 µM α-13’-COOH as described above. After pre-incubation, cells were further stimulated with either 2 ng (low) or 20 ng (high) HDM extract (BAG-D1, Baselstrasse, Bühlmann Laboratories, Schönenbuch, Switzerland), the positive control, or buffer as control (50 min, 37 °C). sLT concentrations were quantified as described in the first experiment. 

### 4.11. Determination of Basophil Activation

To investigate whether α-13’-COOH has an effect on basophil activation itself, the basophil activation test kit (Flow CAST; Bühlmann Laboratories, Baselstrasse, Schönenbuch, Switzerland) was conducted. α-13’-COOH diluted in stimulation buffer was mixed with whole blood to a final concentration of 0.5 and 5 µM. Blood diluted with buffer was used as untreated control. After 50 min of incubation at 37 °C, the pre-incubated blood was mixed with either the specific allergen (HDM extract), fMLP (5 µM), C5a (10 nM), anti-FcεRI mAb (positive control) or buffer as control in falcon tubes. A staining reagent was added to all tubes, composed of a mixture of monoclonal antibodies to human CD63 (anti-CD63-FITC) and human CCR3 (anti-CCR3-PE). All the following steps were conducted according to the manufacturers’ protocols. Samples were measured by flow cytometry (BD FACS Canto; BD BioSciences, Tullastrasse, Heidelberg, Germany).

### 4.12. Histological Preparation, Immunohistochemical Staining and Skin Permeability

Histological and immunohistochemical analyses were carried out as previously described [70,73]. Photographs were taken with a digital camera (AxioCam MRc, Carl Zeiss, Jena, Germany). Evaluation of the thickening of the stratum corneum and filaggrin staining was evaluated by the image processing program ImageJ version v1.53t [74].

Permeability determination was carried out with the fluorescence dye Lucifer Yellow as previously described by Fink et al. [70]. Cell cores were stained with 4′,6-diamidino-2-phenylindole (Sigma Aldrich, Frankfurter Strasse, Darmstadt, Germany). Microscopic assessment was carried out on an Axio Scope A.1 microscope using the FITC filter set at a wavelength of 488 nm. For documentation, photographs were taken with a digital camera AxioCam MRc. 

### 4.13. Statistical Analyses

Data are presented as means ± standard error of the mean. Two independent experiments were performed as replicates with two technical replicates per sample. Histological analyses were conducted on three skin models from two independent experiments with four images per skin model. Scratch assays were evaluated using six images from each sample. Statistical evaluation was performed using GraphPad version 9 (GraphPrism Software, San Diego, CA, USA). One-way ANOVA with multiple comparisons followed by the Tukey post hoc test was conducted. Outliers were detected by the Peirce’s criterion method. Statistical significance was tested with * *p* < 0.05, ** *p* < 0.01 and *** *p* < 0.001. Asterisks [*] indicate significant deviations from the control.

## 5. Conclusions

In summary, our study revealed that histamine enhances migration and proliferation in a scratch closure model as well as inflammation in keratinocytes and full-thickness skin models. Next, we have shown for the first time that histamine triggers hyperkeratosis in atopic dermatitis skin models cultivated with T_H_2 cytokines. We also report new data on the α-tocopherol-derived long-chain metabolite α-13’-COOH, which reduces specific IgE-mediated reactions and de novo synthesis of sLT in vitro. Furthermore, we confirmed the anti-inflammatory properties of α-13’-COOH in a full-thickness atopic dermatitis skin model and provide evidence for the strengthening effects of α-13’-COOH on the skin barrier in this model.

## Figures and Tables

**Table 1:** Primer sequences used for RT-qPCR.

mRNA	Forward Primers (5′ → 3′)	Reverse Primers (5′ → 3′)
CK10	GGGACCAAGATACTAACAAAACC	TGAAAGAACTCTACCGTCGGG
H1R	AAGTCACCATCCCAAACCCCCAAG	TCAGGCCCTGCTCATCTGTCTTGA
H4R	CCGTTTGGGTGCTGGCCTTCTTAG	GATCACGCTTCCACAGGCTCCAAT
NELL2	AGCCAAAACATCAGCCAAGC	TTCCCTTCATGGTGCAAGTC
HAS3	TCCACACGGAAAAGCACTAC	TGCTCCAGGAAGGCAAAAAG

**Table 2:** Primers used for RT-qPCR.

mRNA	GeneGlobe ID
β-actin	QT01680476
FLG	QT00092218
IVL	QT00082586
CCL26	QT00023135
CA2	QT00031059

## Data Availability

All data underlying the results are available as part of the article and no additional source data are required. Further inquiries can be directed to the corresponding author.
